# Paediatric dengue infection in Cirebon, Indonesia: a temporal and spatial analysis of notified dengue incidence to inform surveillance

**DOI:** 10.1186/s13071-019-3446-3

**Published:** 2019-04-29

**Authors:** Endang Puji Astuti, Pandji Wibawa Dhewantara, Heni Prasetyowati, Mara Ipa, Cucu Herawati, Kadina Hendrayana

**Affiliations:** 1 Pangandaran Unit for Health Research and Development, National Institute of Health Research and Development, Ministry of Health of Indonesia, Pangandaran, West Java Indonesia; 2 UQ Spatial Epidemiology Laboratory, School of Veterinary Science, The University of Queensland, Gatton, QLD 4343 Australia; 3 School of Public Health, Sekolah Tinggi Ilmu Kesehatan (STIKES) Cirebon, Cirebon, West Java Indonesia; 4 Cirebon District Health Office, Cirebon, West Java Indonesia

**Keywords:** Dengue, Spatial analysis, Childhood, Indonesia, GIS, Climate, Temporal

## Abstract

**Background:**

The recent situation of dengue infection in Cirebon district is concerning due to an upsurge trend since the year 2010. The largest dengue outbreak was reported in 2016 which has affected more than 1600 children. A study was conducted to explore the temporal variability of dengue outbreak in Cirebon’s child population in during 2011–2017, and to assess the short-term effects of climatic and environmental factor on dengue incidence. In addition, the spatial pattern of dengue incidence in children and high-risk villages were investigated.

**Methods:**

A total of 4597 confirmed dengue cases in children notified from January 2011 to December 2017 were analysed. Seasonal decomposition analysis was carried out to examine the annual seasonality. A generalized linear model (GLM) was applied to assess the short-term effect of climate and normalized difference vegetation index (NDVI) on dengue incidence. The incidence rate ratio (IRR) of the final model was reported. Spatial analyses were conducted by using Moran’s *I* and local indicator of spatial association (LISA) analyses to explore geographical clustering in incidence and to identify high-risk villages for dengue, respectively.

**Results:**

An annual dengue epidemic period was observed with peaks occurring every January/February. Based on the GLM, temperature at a lag 4 months (IRR = 1.27; 95% confidence interval, 95% CI: 1.22–1.31, *P *< 0.001), rainfall at a lag 2 months (IRR = 0.99, 95% CI: 0.99–0.99, *P *< 0.001), humidity at lag 0 month (IRR = 1.05, 95% CI: 1.04–1.06, *P *< 0.001) and NDVI at a lag 1 month (IRR = 3.07, 95% CI: 1.94–4.86, *P *< 0.001) were associated with dengue incidence in children. The dengue incidence in children was spatially varied and clustered at the village level across Cirebon. During 2011–2017, a total of 38 high-risk villages for dengue were identified, which were mainly located in the northern part of Cirebon.

**Conclusions:**

Seasonal patterns of dengue incidence in children in Cirebon were strongly associated with rainfall, temperature, humidity and NDVI variability, suggesting that climatic and environmental data could be used to help predict dengue outbreaks. Our spatial analysis revealed a clustered pattern in dengue incidence and high-risk villages for dengue across Cirebon, suggesting that effective interventions such as vector surveillance and school-based campaigns should be prioritized around the identified high-risk villages. Temporal and spatial analytical tools could be utilized to support local health authorities to apply timely and targeted public health interventions and help better planning and decision-making in order to minimize the impact of dengue outbreaks.

**Electronic supplementary material:**

The online version of this article (10.1186/s13071-019-3446-3) contains supplementary material, which is available to authorized users.

## Background

Dengue fever (DF) is a serious mosquito-borne viral infection worldwide and it is estimated that 50 million of dengue infections occur each year [1, 2]. Infection is caused by four distinct viruses (DENV 1 to 4) belonging to the family Flaviviridae and is transmitted to humans by *Aedes aegypti* and *Ae. albopictus* mosquitoes [3]. Annually, at least four billion people are at risk of acquiring dengue infection [4] and approximately 214,000 (120,000–299,000) disability-adjusted life-years (DALYs) are lost per year due to DF [5]. The burden is predicted to escalate in the future due to an increase in global human mobility, goods transportation and urbanization rates [6, 7]. Furthermore, climate change is thought to intensify dengue outbreaks and expand the spatial dispersion of the vectors, which could bring DENV from endemic regions to non-endemic regions [8].

The occurrence and behavior of *Aedes* spp. is highly dependent on climatic and environmental conditions, which in turn influence the temporal and geographical distributions of DF [9–12]. It is well known that the life-cycle and population dynamics of *Aedes* spp. are greatly influenced by rainfall and temperature [11, 13, 14]. Rainfall provides abundant waterbodies that allow mosquitoes to breed and complete their immature stages. However, heavy rainfall can wash out the breeding sites and thus impact vector population dynamics [15, 16]. Temperature affects the reproductive cycles of mosquitoes and oviposition rates as well as the incubation periods of viruses [14, 17]. Higher temperatures shorten the duration of extrinsic incubation periods (EIP) and thus can escalate the risk of dengue transmission [12, 18]. Moreover, the distribution and movement of *Aedes* mosquitoes is highly affected by the availability and density of larval breeding sites, built-up environments and vegetated areas close to the human settlement [19, 20].

Dengue in Indonesia was first reported in two major cities on the island of Java in 1968 [21]. Since then, local dengue outbreaks have been reported in all 34 provinces and 514 districts across the archipelago [22], placing DF as an important arboviral disease threat. Indonesia is one of the dengue hyper-endemic countries in Southeast Asia where all four dengue serotypes are circulating, thus leading to a greater risk of infection and higher burdens of disease [2, 5]. In particular, most hospitalizations and mortalities among Indonesian children are primarily caused by DF [23]. More than 80% of Indonesian children aged ten years or more who live in the cities have acquired DF at least once in their lifetime [24]. This highlights the need for better surveillance systems as well as preventive and disease control strategies. Currently, the core of dengue control and prevention programs in Indonesia primarily depends on vector control such as community-based vector monitoring, spraying and public health campaigns.

West Java Province is the most populated province in Indonesia, and dengue is also highly endemic in this province. Since the 2000s, the incidence has dramatically increased from 13/100,000 in 2000 to 79/100,000 in 2016 [25]. All the districts and cities in this province are at high risk for dengue transmission, including Cirebon district. Cirebon district is one of the dengue endemic areas situated in coastal lowland areas in northeast West Java and it serves as one of the major economic hubs for West Java and Central Java. Since 2010, dengue incidence in Cirebon district has been continuously increasing. The largest outbreak hit the district in 2016, which affected more than 1600 children [26]. Despite its public health importance, knowledge regarding the temporal pattern of dengue epidemics as well as the geographical distribution of dengue incidence among children across Cirebon district is lacking. Moreover, a full understanding of the factors that influence the temporal and spatial dynamics of dengue among children in this region is far from clear.

Several studies have used spatial analytical approaches to understand the distribution and to identify the areas most affected by dengue infection [27–29]. These studies have shown that geographical information systems (GIS) techniques could provide helpful evidence for designing and implementing surveillance and targeted control measures. A recent study demonstrated evidence for spatial variation in dengue seroprevalence among Indonesian children at the national level [30]. However, this study did not clearly visualize and capture the variation within districts and was limited to urban settings. In this study, we extended knowledge regarding the geographical variability in dengue infection in children at the village level in Cirebon district. This evidence is nonetheless crucial to develop coordinated and evidence-based intervention strategies.

The present study had the following objectives: first, we used temporal modelling approaches to explore the seasonality of dengue incidence among children and to examine the short-term effects of climate and environmental factors on dengue incidence. Secondly, we used spatial analytical tools to investigate the spatial pattern of dengue incidence in children and to identify high-risk villages across the Cirebon district. The results of this study will be beneficial for better informing local health authorities in anticipating dengue outbreaks as well as planning and implementing timely targeted intervention programs (e.g. health promotions and vector control).

## Methods

### Study area

**Fig. 1:**
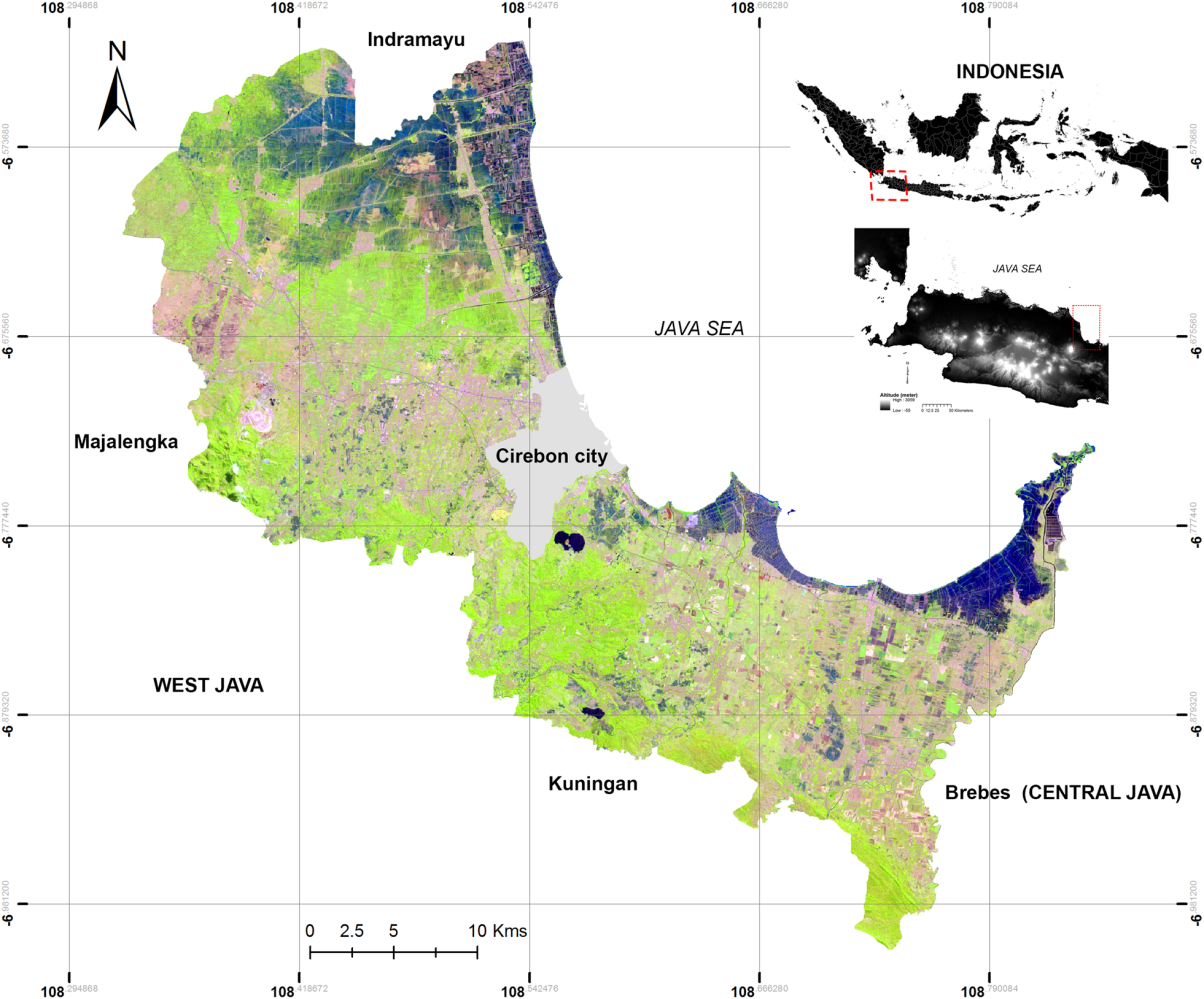
Study site. Satellite image was obtained from Sentinel-2 satellite (https://landsatlook.usgs.gov/sentinel2/viewer.html). Visualization was created in ArcGIS v.10.5 software (Environmental Systems Research Institute, Inc., Redlands, CA, USA)

The district of Cirebon is one of 114 districts/cities in the Java islands and is located in the northwest West Java province about 200 km from the capital Bandung (Fig. 1). The district encompasses 40 ‘*kecamatans*’ (administrative level-2) and 424 villages (administrative level-3), with a total population of approximately 2.3 million people and covering an area of 990 km^2^. Cirebon has a tropical climate with annual mean precipitation of 2575 mm, temperatures ranging between 22–32 °C and a relatively short dry season (June to October) [31]. About 90% of the area is lowland (11–130 m above sea level); the higher altitudes are located in the southwest of the district (Additional file 1: Figure S1).

### Data collection

#### Dengue data

Our analysis was restricted to all confirmed dengue infection [including dengue fever (DF), dengue haemorrhagic fever (DHF) and dengue shock syndrome (DSS)] data in children aged 0–19 years-old that were notified during the period January 1st 2011 to December 31st 2017. We obtained the dataset as an electronic spreadsheet from the Cirebon District Health Office. Since 1968, dengue infection has been a notifiable disease in Indonesia, which means that all dengue cases captured by hospitals must be reported to the District Health Office (DHO) within 24 h of diagnosis using a standardized reporting form. These notified cases are validated through epidemiological investigations by local health authorities. These data contained information including patient age, patient gender, date of onset, patient address (village and subdistrict ID), and diagnosis (suspected, DF, DHF or DSS). All cases of dengue infection were diagnosed according to the nationally standardized diagnostic criteria issued by the Ministry of Health of Indonesia. DF is defined when the patient presents with fever and with two or more of the following symptoms: headache, retro-orbital pain, myalgia, arthralgia, rash, haemorrhagic manifestations and no evidence of plasma leakage with positive leucopenia [≤ 5000 cells/mm^3^, thrombocytopenia (platelet count < 150,000 cells/mm^3^)], an increase in haematocrit (5–10%) and no evidence of plasma loss. DHF is defined as having at least the first two of the following four clinical manifestations: sudden onset acute fever of 2–7 days duration, spontaneous haemorrhagic manifestations or a positive tourniquet test, hepatomegaly, and circulatory failure, in combination with haematological criteria of thrombocytopenia (< 100,000 cells/mm^3^) and ≥  20% increased haematocrit. DSS is defined as DHF plus a rapid, weak pulse with narrow pulse pressure (≤ 20 mmHg), hypotension with cold, clammy skin, and restlessness [2, 32, 33]. The confirmation of dengue infection was based either on a positive anti-dengue virus IgM in acute or convalescent serum samples and/or a 4-fold increase in specific IgG antibody titres between the acute and the convalescent samples or virus by isolation or detection of dengue antigen or RNA in serum [32, 33]. For the purpose of analysis, we aggregated cases for all three outcomes (DF, DHF and DSS) by month.

#### Social, environmental and climate data

Village-level population and population density data were collected from the Cirebon Bureau of Statistics (https://cirebonkab.bps.go.id/publication.html). Environmental data including elevation and normalized difference vegetation index (NDVI) were obtained from satellite imagery data. Elevation data was extracted from the Shuttle Radar Topography Mission (SRTM) at ~ 30 m spatial resolution from the USGS EROS Archive (https://eros.usgs.gov/). Monthly MODIS 13A1 NDVI raster data with a 16-day composite and 500-m spatial resolution for the period January 2011 to December 2017 were also collected. NDVI indicates vegetation greenness and is frequently used as a proxy for the presence of mosquito-favoured habitat [34–36]. Additionally, daily meteorological measures (precipitation, humidity and temperature) for the same period were obtained from the Meteorological, Climatological and Geophysical Bureau database (http://dataonline.bmkg.go.id/). Prior to the analysis, these daily meteorological data were averaged into monthly windows.

### Data analysis

#### Temporal distribution of dengue

Monthly numbers of confirmed dengue infection (aggregated numbers for DF, DHF and DSS) in children by age-group (categorized as either under 5 years or adolescent aged 5 to 19 years old), gender, and season were summarized. Boxplots of seasonal distributions of dengue incidence was also produced. In addition, a seasonal decomposition analysis with Loess (STL) smoothing [37] was performed to explore seasonal patterns and trends in dengue incidence in children using functions from the *stlplus* package in the R statistical project (R v.3.5.0, R Development Core Team, 2017). This analysis decomposes time-series data to generate the trend component (T_*t*_) at time *t*, a cyclical component (C_*t*_) at time *t*, a seasonal component (S_*t*_) at time *t* [which we herein refer to as a seasonal factor (SAF)] and an irregular (remainder) (I_*t*_) component at time *t* [37].

#### Associations between environmental factors (climate, NDVI) and dengue incidence

The relationships between monthly dengue incidence and environmental factors were examined. Monthly dengue counts were defined as dependent variables. Four independent variables including mean temperature, humidity, rainfall and NDVI were included. A Spearman’s correlation test was performed to examine possible associations between covariates. Strongly correlated variables (Spearman’s rho |≥ |0.9|) were excluded to avoid collinearity issues. Associations were considered statistically significant at *P* < 0.05. A cross-correlation analysis was then performed to investigate significant temporal lags (0 to 7 months) between dengue incidence and the remaining covariates. Variables that did not show a significant temporal lag were not included in the final model. We included all covariates that reached positive and negative significant lag values in the model selection processes with a maximum lag of seven months, according to previous findings [9, 38] and the biological and epidemiological plausibility of dengue transmission. In addition, based on the decomposition analysis where a strong seasonality was observed in the data (see Results), the seasonal component or seasonal factor (SAF) obtained from the decomposition analysis was included in the regression model to control the effect of seasonality in the model [39]. To assess the short-term effects of climate and NDVI on dengue incidence in children, a Poisson generalized linear model (GLM) model with log-link was applied. The natural logarithm of the human population was added as an offset term. The most parsimonious model was chosen according to the Akaike information criterion (AIC) value; the model with the lowest AIC was selected as the best-fit model [40]. The presence of seasonality and autocorrelations of the residuals were checked by visually examining the sequence charts and partial autocorrelation function (PACF) plots over time lags. All statistical analyses were conducted using STATA v.15.1 (STATA Corp., College Station, TX, USA).

#### Mapping, spatial clustering and hotspot detection

In this study, we used the village (*n* = 424) as the spatial unit of analysis. All dengue cases were linked with village ID and polygons in ArcGIS v.10.5 (Environmental Systems Research Institute, Inc., Redlands, CA, USA). A village-level boundary map for the Cirebon district was obtained from the National Bureau of Statistics (*Sistem Informasi Layanan Statistik*) (https://www.bps.go.id/).

Using village-level population data as the denominator, the crude incidence of dengue in children at the village level was calculated and mapped. Spatial smoothing was applied to reduce extreme variation between villages due to small populations and to help identify possible high-risk villages which that could not be observed using the raw data [41]. A queen-based spatial contiguity weight matrix (where the village polygon shares a common edge or vertex) was constructed. Empirical Bayes (EB) smoothing was performed using spatial empirical tools in the GeoDa v.1.8 software. The smoothed EB rate of dengue was calculated from the total number of cases in a village divided by the total number of people at risk within the village [42].

**Fig. 2:**
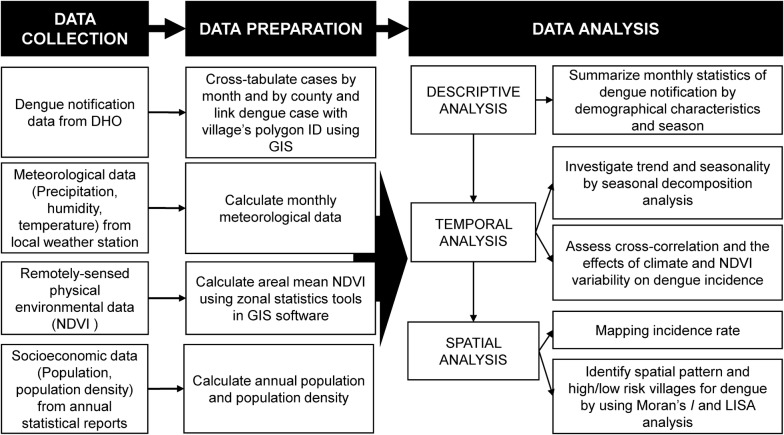
Flowchart of data analysis

To assess the spatial autocorrelation of the EB rate of dengue in children across Cirebon during 2011–2017, a Moran’s *I* analysis was carried out. The values of Moran’s *I* range from − 1 to 1; positive values indicate positive spatial autocorrelation, negative values indicate negative spatial autocorrelation, while values near zero mean the data is randomly distributed [43]. Furthermore, a local indicator spatial association (LISA) analysis was performed to define types of village into categories including high-high (HH) counties (later stated as high-risk counties) or low-low (LL) counties (later stated as low-risk counties) [44]. The high-high counties indicate villages with high rates that are also adjacent to other high-rate villages. Whereas low-low villages have low rates and are close other low-rate villages. Both Moran’s *I* and LISA analyses were also performed by using GeoDA v.1.8 software [45]. All maps were created by using ArcGIS v.10.5.1. An overview of the analytical processes is depicted in Fig. 2.

## Results

### Descriptive statistics

There was a total of 4597 laboratory confirmed dengue infections in children reported from January 2011 to December 2017, which accounted for 80.39% of total notified confirmed dengue cases (*n* = 4597/5718) in Cirebon (Table 1, Additional file 2: Table S1). Of which, 92.47% (*n* = 4251/4597) cases were classified as DHF (Additional file 2: Table S2). The monthly number of dengue cases in children ranged from 0 to 210 cases (mean = 55.38, standard deviation, SD = 51.24); the highest number of cases reported was 210 cases in February 2016. Most dengue cases were observed among children aged 6 to 19 years old (*n* = 3678/4597, 80.00%). Across genders, the proportion of dengue cases was relatively equal. During the rainy season (November to May), the number of notified dengue infections was two-fold higher than those reported during the dry season (June to October).

**Table 1:** Monthly number of notified confirmed dengue cases in children (*N* = 4597), Cirebon, 2011–2017

Characteristic	Frequency	Range	Mean ± SE
Dengue cases	4597	0–210	55.38 ± 51.24
Age (years)			
Under 5	919	0–53	11.48 ± 11.67
6–19	3678	0–167	44.31 ± 40.14
Gender			
Male	2340	0–120	28.53 ± 25.80
Female	2257	0–106	27.52 ± 25.87
Season			
Wet (November–May)	3155	0–264	81.95 ± 68.50
Dry (June–October)	1442	0–141	51.80 ± 37.80

*Abbreviation*: SD, standard deviation

### Temporal trends and seasonality

**Fig. 3:**
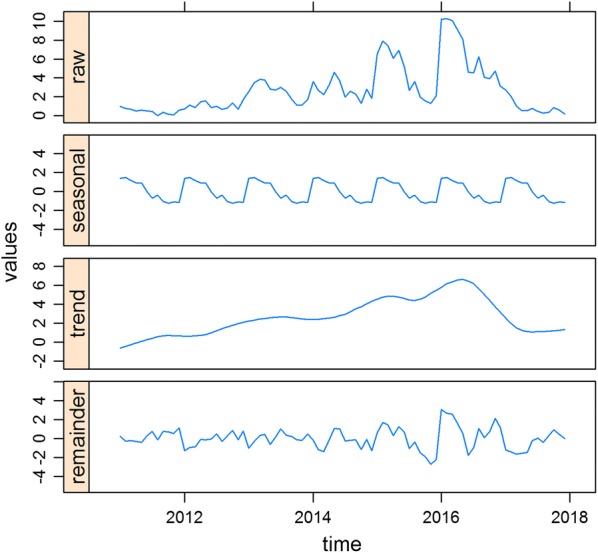
Seasonal decomposition plot of notified dengue in children in Cirebon

**Fig. 4:**
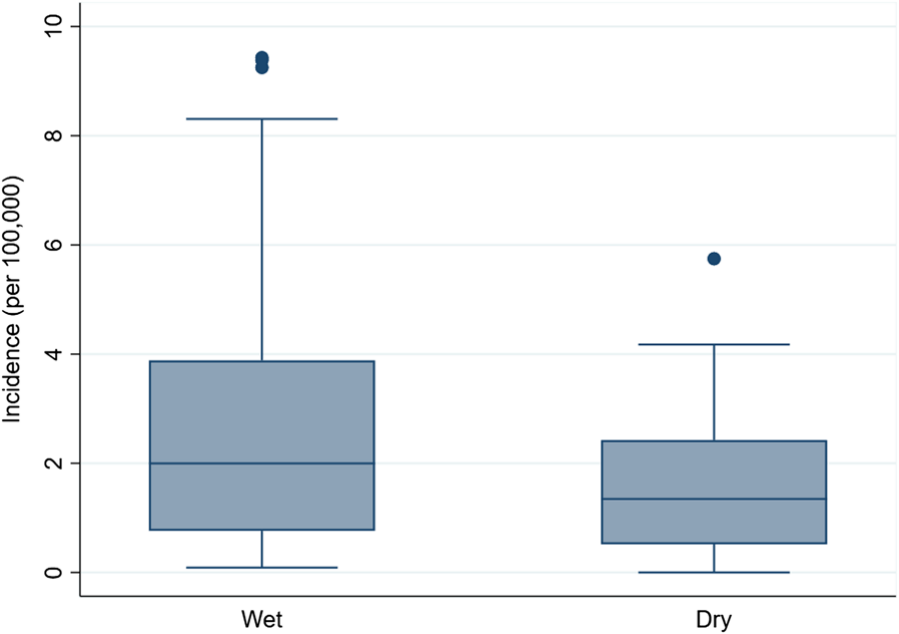
Monthly dengue incidence in children by season, Cirebon

The incidence of dengue in children dramatically increased from 2011 to 2016 but later declined in 2017. In addition, our seasonal decomposition plot demonstrated strong seasonality, with the highest peaks consistently occurring in January/February (Fig. 3). The mean monthly incidence of dengue during the wet season was higher compared to the dry season (Fig. 4).

### Temporal associations between climate, NDVI and dengue incidence

Factors driving the temporal variability of childhood dengue incidence were then investigated. Spearmanʼs correlation analysis demonstrated no strong correlation (Spearman’s rho |≥ |0.9|) between covariates (Additional file 2: Table S3). Thus, we included all covariates in the model selection process. Cross-correlation analysis indicated a positive significant correlation between dengue with rainfall (lag 1–2 months), temperature (lag 4–6 months), humidity (lag 1–2 months) and NDVI (lag 0) (Additional file 3: Figure S2). As we identified strong seasonality in the data, we incorporated the seasonality factor (SAF) in the regression model to adjust for seasonal effects [39]. A total of 18 models were constructed for variable selection (Additional file 2: Table S4). Table 2 shows the parameter estimates of the best-fit generalized linear model (GLM) (AIC = 2483.33). No multicollinearity issues were detected in the final model (mean variance inflation factor, VIF = 1.11). The final model indicated that an increase in temperature at a lag 4 months, humidity in the present month and NDVI at a lag 1 month were associated with a 1.27-fold (95% CI: 1.22–1.31, *P *< 0.001), 1.05-fold (95% CI: 1.04–1.06, *P *< 0.001) and a 3.07-fold (95% CI: 1.94–4.86, *P *< 0.001) increase in dengue incidence rates in children, respectively. Conversely, an increase in rainfall was significantly associated with a slight decrease in childhood dengue incidence by 1% (*P *< 0.001) in the following 2 months.

**Table 2:** Results of generalized linear models for the associations between dengue incidence in children and environmental factors

Variable	Lag (month)	IRR (95% CI)	*P*-value
SAF	0	1.60 (1.43–1.80)	< 0.001
Tmean	4	1.27 (1.22–1.31)	< 0.001
Precipitation	2	0.99 (0.99–0.99)	< 0.001
RH	0	1.05 (1.04–1.06)	< 0.001
NDVI	1	3.07 (1.94–4.86)	< 0.001

*Abbreviations*: IRR, incidence rate ratio; 95% CI, 95% confidence interval; SAF, seasonality factor; RH, relative humidity; NDVI, normalized difference vegetation index

### Spatial clustering and hotspots of dengue

**Fig. 5:**
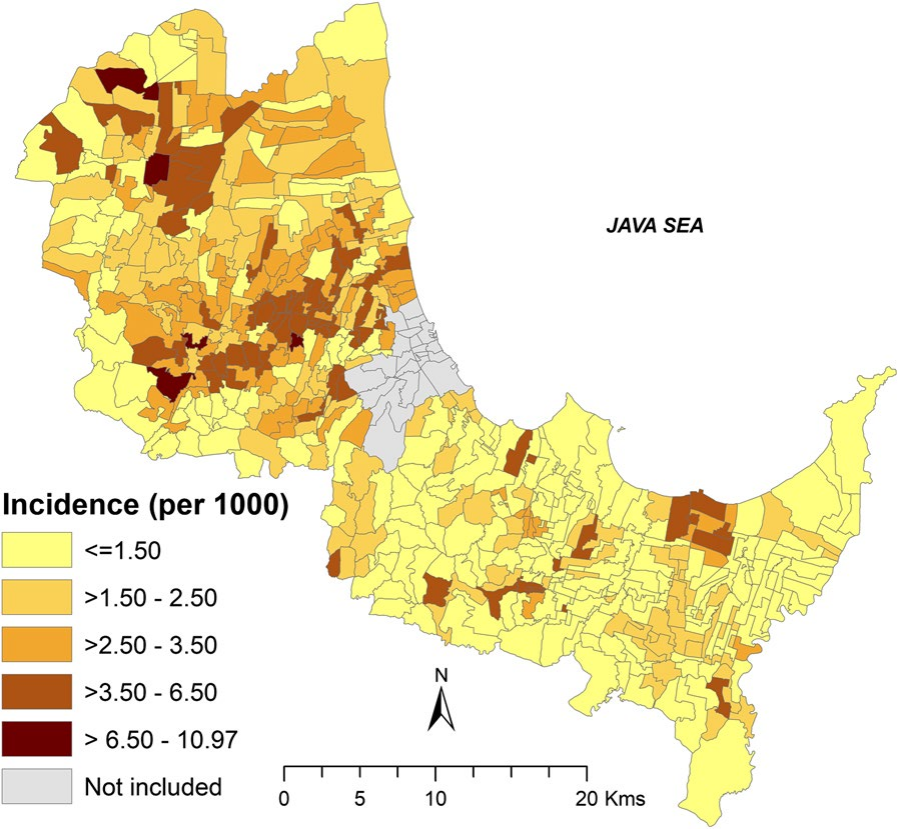
Crude dengue incidence in children (per 1000 population) at village-level in Cirebon District (2010–2017)

In general, the distribution of dengue incidence in children was spatially variable at the village level across Cirebon (Fig. 5), with an incidence ranging between 0–10.97 per 1000 people. Villages with high dengue incidence were mostly observed in the northern part of Cirebon including Plumbon, Kepuh, Kalideres, Kaliwedi and Bode Lor. In the southeast, high incidence villages were Wayanasa, Kanci, Panongan, Susukan Lebak and some villages in the *kecamatan* Gebang. Furthermore, the geographical distribution of childhood dengue infection by age group was also geographically heterogeneous at village-level across Cirebon (Additional file 4: Figure S3).

The results of the Moran’s *I* statistics of smoothed EB dengue rates in children are summarized in Table 3. In general, positively significant Moran’s *I* coefficients (*P *= 0.05) were observed during 2011–2017, except in the year 2012 (which was not statistically significant; *P *= 0.107). The value of *I* fluctuated over time, with the highest coefficient estimated in 2016 (*I* = 0.206, *P *= 0.001) and the lowest coefficient estimated in 2017 (*I *= 0.063, *P *= 0.001).

**Table 3:** Moran’s *I* of the smoothed empirical Bayes (EB) rates of dengue in children, Cirebon, 2011–2017

Year	Moran’s *I*	*P*-value
2011	0.058	0.027
2012	0.034	0.107
2013	0.167	0.001
2014	0.138	0.001
2015	0.199	0.001
2016	0.206	0.001
2017	0.063	0.005
Total	0.314	0.001

During the study period, the LISA analysis identified a total of 38 high-high (HH) villages for dengue. These villages were distributed across 11 *kecamatans*, including Sumber (1 village), Plered (5 villages), Plumbon (10 villages), Palimanan (5 villages), Depok (4 villages), Weru (4 villages), Jamblang (2 villages), Pangurangan (1 village), Gempol (1 village), Gegesik (4 villages) and Klangenan (1 village) (Additional file 2: Table S5).

**Fig. 6:**
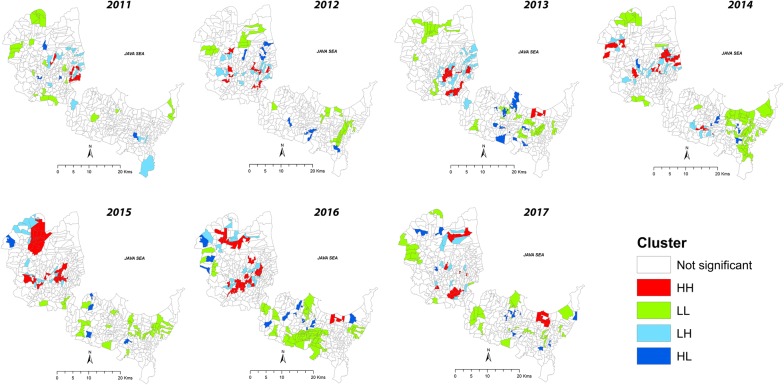
Spatiotemporal clusters of dengue in children in Cirebon as identified by LISA, 2011–2017

Table 4 shows the annual spatial cluster characteristics as identified by LISA. The number of high-risk villages appeared to increase gradually each year, with the highest number of high-risk villages observed in 2016 (30 villages). The total population of children at risk during 2016 was 51,405. The spatiotemporal turnover of high-risk villages for dengue in children during the study period is depicted in Fig. 6. The high-risk counties were predominantly found in the northern part of the district, while the majority of low-low (LL) or low risk villages were situated in the southern part of Cirebon. The maps indicated a dynamic spread of high-risk counties from a small set of high-risk clusters in 2011 around the border between Cirebon district and Cirebon city toward the west from 2012 to 2016. Two pockets of high-risk villages in the north were estimated: one encompassing *kecamatan* Kedawung in the eastern border toward Cirebon city in the west and another encompassing villages in *kecamatan* Gegesik that border the Indramayu district. From 2015–2016, a large group of high-risk counties was identified in the north which also covered the *kecamatan* Gegesik. However, we only found small groups of high-risk villages in the south (belonging to *kecamatan* Gebang) in 2016–2017.

**Table 4:** Characteristics of spatial clusters of dengue in children detected by LISA in Cirebon, 2011–2017

Year	Type of cluster	No. of counties	Population at risk	Child population at risk
2011	HH	7	60,025	15,861
	LL	21	109,881	41,692
	LH	16	58,645	31,451
	HL	4	40,395	5674
2012	HH	11	64,064	23,725
	LL	27	138,883	48,905
	LH	15	108,208	30,068
	HL	7	36,820	9327
2013	HH	18	102,564	32,390
	LL	34	154,251	53,150
	LH	19	104,020	35,805
	HL	8	37,196	14,055
2014	HH	17	98,454	33,798
	LL	43	222,546	69,481
	LH	24	105,767	36,644
	HL	4	23,504	7139
2015	HH	24	146,771	47,449
	LL	30	149,234	48,516
	LH	14	76,654	23,753
	HL	5	28,820	10,137
2016	HH	30	161,616	51,405
	LL	46	202,027	70,134
	LH	17	103,589	37,786
	HL	9	53,761	20,414
2017	HH	16	102,271	38,874
	LL	26	130,744	47,286
	LH	11	60,789	20,183
	HL	9	45,672	16,863

## Discussion

Dengue fever is an important arboviral disease in Indonesia and it is known as an infectious disease that often affects Indonesian children [46]. In this ecological study, we analysed the most recent dengue infection notification records and specifically focused on describing the temporal patterns of childhood dengue infection and exploring climatic and environmental factors associated with its seasonality. In addition, we explored the spatial distribution of dengue incidence in children and investigated high-risk villages across the Cirebon district from 2011–2017.

The trend of notified dengue incidence among the childhood population dramatically increased in Cirebon from 2011–2016 with a large outbreak occurring in 2015. This trend might be driven by a multifactorial process that includes socio-demographic factors, urbanization, environmental changes, human travel and trade that takes place in the area. The district is adjacent to the Cirebon metropolitan area where most of the region’s economic activities happen. The district also lies on the national main roads (e.g. ‘*Jalur Pantai Utara*’ or Northern Coast Mainroad) which connect main cities in the northern coast of the Java islands from Jakarta towards Surabaya. This coastal region is an important hub for industries and for the economies of West Java and Central Java. Economic expansion might have led to rapid urbanization as well as environmental changes that could have facilitated the expansions of vectors and the importation of viruses, which in turn could have driven the temporal and spatial distribution of dengue incidence in Cirebon district. The increasing trend in dengue incidence that our study shows suggests that steps to improve dengue surveillance systems in this area should be taken immediately.

Dengue in Cirebon is highly seasonal, with peaks in incidence taking place in January or February, which clearly coincides with the annual rainy season. This finding is consistent with reports from other parts of Indonesia [38, 47], which also demonstrated that the majority of dengue infection occurs during the rainy season from January to April. The present study confirmed that the incidence rate was higher during the wet season compare to the dry season, highlighting that climate plays an important role in dengue transmission in this region.

To further clarify factors associated with the temporal pattern of dengue incidence, we assessed the relationship between rainfall, temperature, humidity and NDVI variability and monthly dengue incidence. Effects of rainfall and temperature variability on dengue incidence have been extensively studied in many locations in Indonesia [38, 48, 49]; however, no study so far has paid much attention to their associations with dengue incidence in children, especially in Cirebon. In this study, we found positive correlations for temperature, humidity and NDVI with childhood dengue incidence. We found that an increase in temperature could double dengue incidence in children in the following two to four months, while a one unit increase in humidity in the present month is likely to increase dengue incidence by 5%. Indeed, these findings indicated the significant role of such factors in vector development. These findings are consistent with previous results [10, 38, 50]. For instance, Tosepu et al. [38], working in Kendari city in southeast Sulawesi, also found a positive correlation between temperature (at a lag 2 months) and dengue incidence. Temperature and humidity have been considered as important factors for mosquito biology, population dynamics, survival as well as capacity to transmit dengue virus [11, 51]. *Aedes* spp. require optimum temperatures ranging between 15–35 °C to fully develop and survive. Moreover, studies by Chen & Hsieh [52] in Taiwan and Sánchez-González et al. [53] in Mexico indicated that the highest risk of dengue transmission generally occurs at a temperature of 28 °C. There is compelling evidence that this optimal temperature reduced gonotrophic cycles for *Aedes* mosquitoes, prolonging of the mosquitoes’ life expectancy, shortening of the extrinsic incubation periods of arboviruses, and facilitating the dispersal of vectors across broader geographical areas [13, 14, 51], which could potentially lead to a greater probability of dengue transmission. However, temperature and humidity are not the only climate factors influencing *Ae. aegypti* existence and dengue occurrence.

Interestingly, we found a negative association between rainfall and dengue incidence in the following two months, although the effect was relatively weak. Similar results has also been reported by a study in Guangzhou [54], but our finding was in disagreement with a study conducted in Yogyakarta [49] which found a positive correlation between rainfall and dengue incidence. It is known that rainfall can provide considerable amounts of waterbodies which allow mosquitoes to breed and complete their immature stage and thus could increase mosquito population in the environment. However, high rainfall intensity may also cause flash flooding that could flush out breeding sites and thus decrease the vector population, which in turn could limit the potential for dengue transmission [15].

Our study also demonstrated a strong association between vegetation greenness and monthly dengue incidence, which also supports findings from other studies [19, 35, 36]. Our study demonstrated that the greater the estimated NDVI, the higher incidence rate of dengue in the following month. NDVI has been found to act as a biological index of environmental conditions influencing the population dynamics of *Aedes* mosquitoes [55–57]. The greener vegetation suggests that there were more favourable habitats for mosquitoes which help maintain vector populations in the environment [58]. Indeed, as shown in the satellite image (Fig. 1), residential areas are predominantly situated in peri-urban areas and are surrounded by vegetated areas (e.g. rice fields, shrubs) which are known to be favourable habitats for mosquitoes such as *Ae. albopictus*. These environmental conditions could influence the dispersal and movement of vectors [18–20]. In fact, children mostly spend their days in school in the morning and but at playgrounds in the afternoon, which coincides with the peak biting activity of mosquitoes. This active behaviour as well as their vulnerable immune levels indeed puts children at a higher risk of becoming infected, especially during the wet season when their immunity profiles commonly reduce while at the same time breeding sites and *Aedes* populations are abundant, leading to a high humans-vector contact rate. Based on our evidence, we recommend that preventive efforts such as school-based health campaigns and environmental manipulation to reduce breeding sites around the schools and parks should be strengthened, especially in the month or two prior to the beginning of wet season. Our identified links between peaks in dengue incidence and temperature, rainfall, humidity and NDVI ranging from one to four months lag could be explained by the mechanisms of dengue transmission that are ongoing. Our finding is consistent with the probable mechanism of dengue transmission. To illustrate, *Ae. aegypti* mosquitoes require two weeks to fully complete their life-cycle from eggs to adults. The females’ extrinsic incubation period (EIP) can last from 8 to 12 days depending on the temperature and virus type [59, 60] and, on average, the intrinsic incubation period for dengue ranges from one to two weeks. Breeding sites both inside and outside the households were abundant during both the dry and wet season. In the dry season people tend to store water inside which facilitates *Ae. aegypti* in laying their eggs. Once the rainy season begins, vegetation becomes dense and the large amount of uncontrolled water containers outside (e.g. used tyres, tins, bottles) allow *Ae. albopictus* to complete their cycle.

Spatial analysis allows the visualization of villages with the highest dengue incidence as well as exploration of its geographical pattern over time. In this study, we revealed that the dengue incidence in children was spatially clustered, except in the year 2012. Furthermore, we identified that most high-risk villages were located in the northern part of Cirebon and in close proximity to the main road networks that connect Cirebon with Majalengka and Indramayu. These two district areas are also known as dengue-prone areas. This finding corroborates studies elsewhere [36, 61, 62] which found strong relationships between road networks and high-risk areas of dengue. One explanation for this is that incidence may be associated with the social and environmental features of this area. To illustrate, most dense and urbanized areas in Cirebon are found in the northern part of the district, which is surrounded by vegetated land. In addition, most inhabitants often store water in containers inside the house since water supply in this region is poorly distributed especially during the dry season. Such conditions increase the chances of oviposition of *Ae. aegypti* and house-to-house vector dispersal, which leads to increase the spillover of the virus resulting in higher dengue incidence. Moreover, the presence of road networks allows intense human mobility and rapid urbanization that could facilitate DENV spread, contributing to a greater risk of dengue outbreaks. Factors such as human movement, urbanization, landscape characteristics together with favourable climatic factors may greatly escalate the likelihood of contact between humans and vectors and thus increase risk of dengue transmission in this region.

The spatiotemporal maps of high-risk villages provided in this study indicate that local-level factors have driven the dynamics of dengue incidence in children from 2011–2017. Factors including climatic, environmental, socioeconomic conditions as well as control measures could possibly change the spatial distribution of high-risk villages. However, specific factors underlying these spatiotemporal changes are unclear. Therefore, local epidemiological investigations are required to identify drivers at the village level.

There are some limitations in this study that require our findings to be carefully interpreted. First, we analysed passive surveillance dengue data, which may more likely underestimate the actual dengue incidence in children due to underreported cases. However, our findings still provide important information regarding temporal and spatial variability of dengue incidence in children that is beneficial for designing timely and targeted village-level prevention and control measures. Secondly, the temporal and spatial trends in dengue incidence in children as observed in this study might partly be driven by other confounding factors including individual-level (e.g. nutrition status, immunity, behaviour), household-level factors (e.g. socioeconomic, water storage behaviour, vector control practices) and environment at the village level which were not included in this study. Future epidemiological studies should investigate the effect of such socio-ecological factors and control efforts on the geographical and temporal distribution of dengue risk in children.

## Conclusions

To our knowledge, this is the first ecological study investigating temporal and spatial patterns of dengue incidence in children at the village-level in Indonesia. This study showed that dengue infection is common in the childhood population in Cirebon and the trend of incidence is likely to increase over time. Regular outbreaks were strongly linked with local climate and environmental variability. In addition, this study identified clusters of high-risk villages in the north along the main road networks. The results of this study could be utilized to establish a dengue temporal and spatial early warning system, which potentially could support local health authorities to apply timely interventions as well as better planning and decision-making in order to minimize the impact of dengue outbreaks. Our study recommends that strengthening of prevention and control measures for dengue in children should be rely on applying vector control and school-based health campaigns, especially in the identified high-risk villages.

## Additional files

**Additional file 1: Figure S1.** Map of elevation of Cirebon district. Elevation data from Shuttle Radar Topography Mission (SRTM) with ~ 30 m spatial resolution was retrieved from USGS EROS Archive (https://eros.usgs.gov/). About 90% of the area is lowland; the higher altitudes are located in the southwest of the district.

**Additional file 2: Table S1.** Annual reported number of confirmed dengue cases by age-group, Cirebon district, 2011–2017. **Table S2.** Number of DHF, DSS, DD and suspected dengue cases in children, Cirebon district, 2011–2017. **Table S3**. Spearmanʼs correlation between covariates. **Table S4.** Models of associations between climatic factors and NDVI and dengue incidence as identified by a generalized linear model. **Table S5.** High-risk villages (*n* = 38) for dengue in children identified by LISA analysis, Cirebon district, 2011–2017.

**Additional file 3: Figure S2.** Cross-correlation analysis between dengue and rainfall, temperature humidity and NDVI, Cirebon, West Java, Indonesia. Cross-correlation analysis indicated a positive significant correlation between dengue with rainfall (lag 1–2 months), temperature (lag 4–6 months), humidity (lag 1–2 months) and NDVI (lag 0).

**Additional file 4: Figure S3.** Crude incidence of dengue among U5s (A) and adolescents (5–19 years-old) (B) at village level, Cirebon, 2011–2017.

## Abbreviations

DF: dengue fever
DHF: dengue haemorrhagic fever
DSS: dengue shock syndrome
DENV: dengue virus
DHO: District Health Office
GIS: geographical information systems
GLM: generalized linear model
STL: seasonal decomposition analysis with Loess
IRR: incidence rate ratio
LISA: local indicator of spatial association
SRTM: Shuttle Radar Topography Mission
NDVI: normalized difference vegetation index
CI: confidence interval

## References

[1] Gubler DJ (2002). The global emergence/resurgence of arboviral diseases as public health problems. Arch Med Res.

[2] World Health Organization-SEARO (2011). Comprehensive guidelines for prevention and control of dengue and dengue hemorrhagic fever.

[3] Halstead SB (2008). Dengue virus–mosquito interactions. Ann Rev Entomol.

[4] Bhatt S, Gething PW, Brady OJ, Messina JP, Farlow AW, Moyes CL (2013). The global distribution and burden of dengue. Nature.

[5] Shepard DS, Undurraga EA, Halasa YA (2013). Economic and disease burden of dengue in Southeast Asia. PLoS Negl Trop Dis..

[6] Tian H, Sun Z, Faria NR, Yang J, Cazelles B, Huang S (2017). Increasing airline travel may facilitate co-circulation of multiple dengue virus serotypes in Asia. PLoS Negl Trop Dis..

[7] Guzman A, Istúriz RE (2010). Update on the global spread of dengue. Int J Antimicrob Agents.

[8] Kraemer MUG, Sinka ME, Duda KA, Mylne AQN, Shearer FM, Barker CM (2015). The global distribution of the arbovirus vectors *Aedes aegypti* and *Ae. albopictus*. eLife.

[9] Choi Y, Tang CS, McIver L, Hashizume M, Chan V, Abeyasinghe RR (2016). Effects of weather factors on dengue fever incidence and implications for interventions in Cambodia. BMC Public Health..

[10] Wangdi K, Clements ACA, Du T, Nery SV (2018). Spatial and temporal patterns of dengue infections in Timor-Leste, 2005–2013. Parasites Vectors.

[11] Christophers SR (1960). *Aedes aegypti* (L.), the yellow fever mosquito: its life history, bionomics and structure.

[12] Mordecai EA, Cohen JM, Evans MV, Gudapati P, Johnson LR, Lippi CA (2017). Detecting the impact of temperature on transmission of Zika, dengue, and chikungunya using mechanistic models. PLoS Negl Trop Dis..

[13] Reinhold J, Lazzari C, Lahondère C (2018). Effects of the environmental temperature on *Aedes aegypti* and *Aedes albopictus* mosquitoes: a review. Insects..

[14] Xiao FZ, Zhang Y, Deng YQ, He S, Xie HG, Zhou XN, Yan YS (2014). The effect of temperature on the extrinsic incubation period and infection rate of dengue virus serotype 2 infection in *Aedes albopictus*. Arch Virol.

[15] Benedum CM, Seidahmed OME, Eltahir EAB, Markuzon N (2018). Statistical modeling of the effect of rainfall flushing on dengue transmission in Singapore. PLoS Negl Trop Dis..

[16] Koenraadt C, Harrington L (2008). Flushing effect of rain on container-inhabiting mosquitoes *Aedes aegypti* and *Culex pipiens* (Diptera: Culicidae). J Med Entomol.

[17] Delatte H, Gimonneau G, Triboire A, Fontenille D (2009). Influence of temperature on immature development, survival, longevity, fecundity, and gonotrophic cycles of *Aedes albopictus*, vector of chikungunya and dengue in the Indian Ocean. J Med Entomol.

[18] Wong GKL, Jim CY (2016). Do vegetated rooftops attract more mosquitoes? Monitoring disease vector abundance on urban green roofs. Sci Total Environ.

[19] Meza-Ballesta A, Gonima L (2014). The influence of climate and vegetation cover on the occurrence of dengue cases (2001–2010). Rev Salud Publica.

[20] Heinisch MRS, Diaz-Quijano FA, Chiaravalloti-Neto F, Menezes Pancetti FG, Rocha Coelho R, Dos Santos Andrade P (2019). Seasonal and spatial distribution of *Aedes aegypti* and *Aedes albopictus* in a municipal urban park in Sao Paulo, SP, Brazil. Acta Trop.

[21] Sumarmo (1987). Dengue haemorrhagic fever in Indonesia. Southeast Asian J Trop Med Public Health..

[22] Ministry of Health, Republic of Indonesia (2018). Indonesia health profile 2017.

[23] Adrizain R, Setiabudi D, Chairulfatah A (2018). Hospital-based surveillance: accuracy, adequacy, and timeliness of dengue case report in Bandung, West Java, Indonesia of 2015. J Global Infect Dis..

[24] Prayitno A, Taurel AF, Nealon J, Satari HI, Karyanti MR, Sekartini R (2017). Dengue seroprevalence and force of primary infection in a representative population of urban dwelling Indonesian children. PLoS Negl Trop Dis..

[25] Provincial Health Office of West Java. West Java health profile. Bandung: Dinas Kesehatan Provinsi Jawa Barat; 2017. http://www.diskes.jabarprov.go.id/. Accessed 27 Nov 2018.

[26] District Health Office of Cirebon. Cirebon health profile. Cirebon: Dinas Kesehatan Kabupaten Cirebon; 2017. http://dinkes.cirebonkab.go.id/. Accessed 27 Nov 2018.

[27] Dhewantara PW, Ruliansyah A, Fuadiyah ME, Astuti EP, Widawati M (2015). Space-time scan statistics of 2007–2013 dengue incidence in Cimahi City, Indonesia. Geospat Health..

[28] Gil JF, Palacios M, Krolewiecki AJ, Cortada P, Flores R, Jaime C (2016). Spatial spread of dengue in a non-endemic tropical city in northern Argentina. Acta Trop.

[29] Liu C, Liu Q, Lin H, Xin B, Nie J (2014). Spatial analysis of dengue fever in Guangdong Province, China, 2001–2006. Asia Pac J Public Health.

[30] Tam CC, OʼDriscoll M, Taurel AF, Nealon J, Hadinegoro SR (2018). Geographic variation in dengue seroprevalence and force of infection in the urban paediatric population of Indonesia. PLoS Negl Trop Dis..

[31] Bureau of Statistics of Cirebon. Cirebon District in Figure 2017. Cirebon: Bureau of Statistics of Cirebon District; 2018. https://cirebonkab.bps.go.id/publication.html. Accessed 27 Nov 2018.

[32] WHO (1997). Dengue haemorrhagic fever: diagnosis, treatment, prevention and control.

[33] Ministry of Health, Republic of Indonesia (2005). Guidelines on dengue prevention and control in healthcare facilities in Indonesia.

[34] Hayden MH, Uejio CK, Walker K, Ramberg F, Moreno R, Rosales C (2010). Microclimate and human factors in the divergent ecology of *Aedes aegypti* along the Arizona, U.S./Sonora, MX border. EcoHealth..

[35] Huang CC, Tam T, Chern YR, Lung SC, Chen NT, Wu WD (2018). Spatial clustering of dengue fever incidence and its association with surrounding greenness. Int J Environ Res Public Health..

[36] Qi X, Wang Y, Li Y, Meng Y, Chen Q, Ma J, Gao GF (2015). The effects of socioeconomic and environmental factors on the incidence of dengue fever in the Pearl River Delta, China, 2013. PLoS Negl Trop Dis..

[37] Cleveland RB, Cleveland WS, McRae JE, Terpenning I (1990). STL: a seasonal-trend decomposition. J Off Stat.

[38] Tosepu R, Tantrakarnapa K, Nakhapakorn K, Worakhunpiset S (2018). Climate variability and dengue hemorrhagic fever in Southeast Sulawesi Province, Indonesia. Environ Sci Pollut Res..

[39] Kumar A (2010). On the assessment of the value of the seasonal forecast information. Meteorol Appl..

[40] Dupont WD (2009). Statistical modeling for biomedical researchers: a simple introduction to the analysis of complex data.

[41] Hu W, Clements A, Williams G, Tong S (2011). Spatial analysis of notified dengue fever infections. Epidemiol Infect.

[42] Anselin L (2005). Exploring spatial data with GeoDa: a workbook.

[43] Moran PAP (1950). Notes on continuous stochastic phenomena. Biometrika.

[44] Anselin L (1995). Local indicators of spatial association, LISA. Geogr Anal.

[45] Anselin L, Syabri I, Kho Y (2006). GeoDa, an introduction to spatial data analysis. Geogr Anal..

[46] L’Azou M, Moureau A, Sarti E, Nealon J, Zambrano B, Wartel TA (2016). Symptomatic dengue in children in 10 Asian and Latin American countries. N Engl J Med.

[47] Corwin AL, Larasati RP, Bangs MJ, Wuryadi S, Arjoso S, Sukri N (2001). Epidemic dengue transmission in southern Sumatra, Indonesia. Trans R Soc Trop Med Hyg.

[48] Kesetyaningsih TW, Andarini S, Sudarto S, Pramoedyo H (2018). Determination of environmental factors affecting dengue incidence in Sleman, Yogyakarta, Indonesia. Afr J Infect Dis..

[49] Ramadona AL, Lazuardi L, Hii YL, Holmner Å, Kusnanto H, Rocklöv J (2016). Prediction of dengue outbreaks based on disease surveillance and meteorological data. PLoS ONE.

[50] Lover AA, Buchy P, Rachline A, Moniboth D, Huy R, Meng CY (2014). Spatial epidemiology and climatic predictors of paediatric dengue infections captured via sentinel site surveillance, Phnom Penh Cambodia 2011–2012. BMC Public Health..

[51] Morin CW, Comrie AC, Ernst K (2013). Climate and dengue transmission: evidence and implications. Environ Health Perspect.

[52] Chen SC, Hsieh MH (2012). Modeling the transmission dynamics of dengue fever: implications of temperature effects. Sci Total Environ.

[53] Sánchez-González G, Condé R, Noguez Moreno R, López Vázquez PC (2018). Prediction of dengue outbreaks in Mexico based on entomological, meteorological and demographic data. PLoS ONE.

[54] Kong L, Xu C, Mu P, Li J, Qiu S, Wu H (2018). Risk factors spatial-temporal detection for dengue fever in Guangzhou. Epidemiol Infect.

[55] Hira FS, Asad A, Farrah Z, Basit RS, Mehreen F, Muhammad K (2018). Patterns of occurrence of dengue and chikungunya, and spatial distribution of mosquito vector *Aedes albopictus* in Swabi district, Pakistan. Trop Med Int Health.

[56] Scavuzzo JM, Trucco F, Espinosa M, Tauro CB, Abril M, Scavuzzo CM, Frery AC (2018). Modeling dengue vector population using remotely sensed data and machine learning. Acta Trop.

[57] Estallo EL, Lamfri MA, Scavuzzo CM, Almeida FFL, Introini MV, Zaidenberg M, Almirón WR (2008). Models for predicting *Aedes aegypti* larval indices based on satellite images and climatic variables. J Am Mosq Control Assoc..

[58] Medeiros-Sousa AR, Ceretti W, Urbinatti PR, De Carvalho GC, De Paula MB, Fernandes A (2013). Mosquito fauna in municipal parks of São Paulo City, Brazil: a preliminary survey. J Am Mosq Control Assoc..

[59] Watts DM, Burke DS, Harrison BA, Whitmire RE, Nisalak A (1987). Effect of temperature on the vector efficiency of *Aedes aegypti* for dengue 2 virus. Am J Trop Med Hyg.

[60] Salazar MI, Richardson JH, Sanchez-Vargas I, Olson KE, Beaty BJ (2007). Dengue virus type 2: replication and tropisms in orally infected *Aedes aegypti* mosquitoes. BMC Microbiol.

[61] Li Q, Cao W, Ren H, Ji Z, Jiang H (2018). Spatiotemporal responses of dengue fever transmission to the road network in an urban area. Acta Trop.

[62] Mahabir RS, Severson DW, Chadee DD (2012). Impact of road networks on the distribution of dengue fever cases in Trinidad, West Indies. Acta Trop.

