# Supplementary figures and images for: Paediatric dengue infection in Cirebon, Indonesia: a temporal and spatial analysis of notified dengue incidence to inform surveillance
Source: Parasit Vectors. 2019 Apr 29;12:186. doi: 10.1186/s13071-019-3446-3 (PMC6489314; doi:10.1186/s13071-019-3446-3)

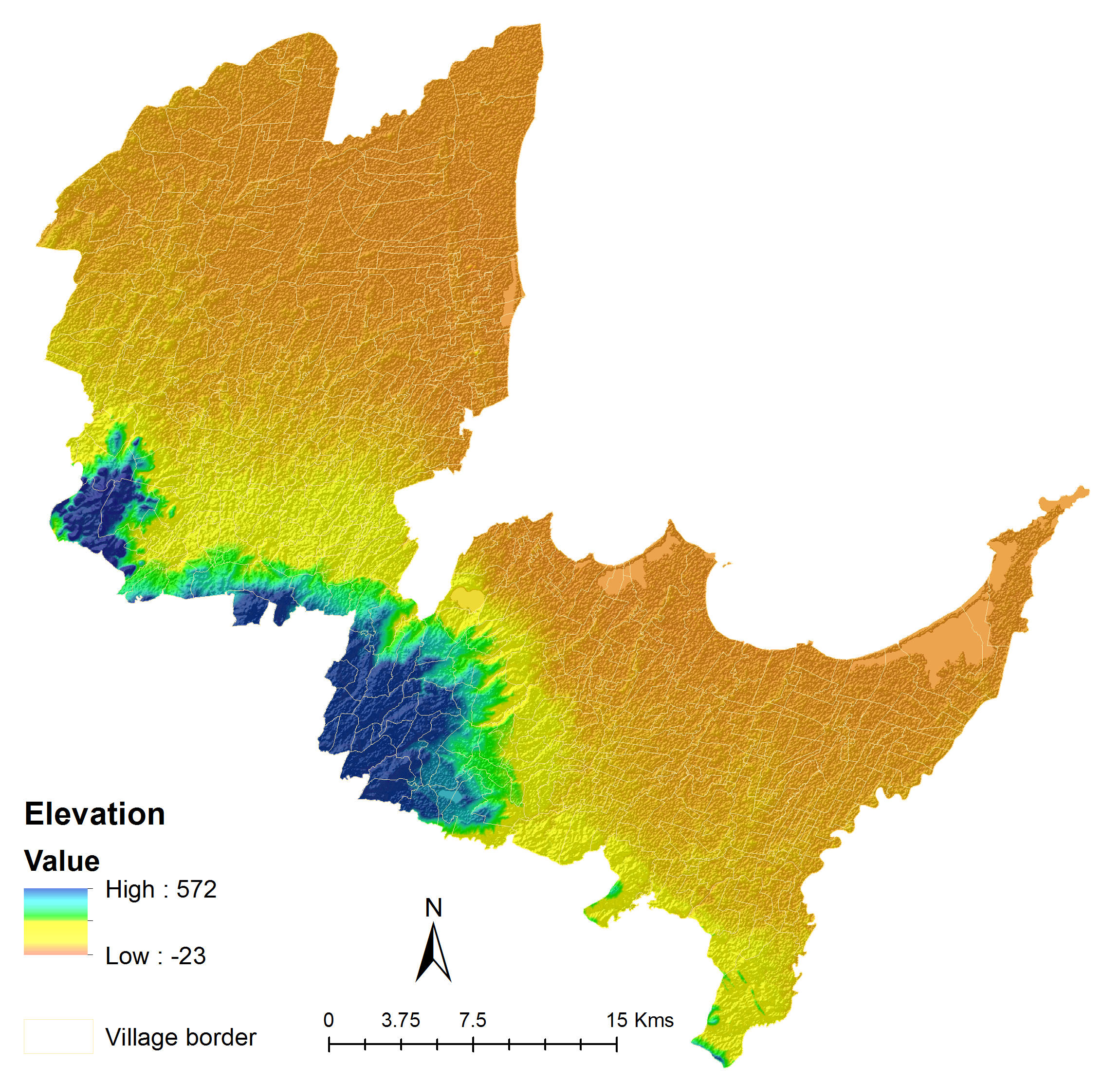

Supplement: Supplementary file 1 — Additional file 1: Figure S1. Map of elevation of Cirebon district. Elevation data from Shuttle Radar Topography Mission (SRTM) with ~ 30 m spatial resolution was retrieved from USGS EROS Archive (https://eros.usgs.gov/). About 90% of the area is lowland; the higher altitudes are located in the southwest of the district. [file 13071_2019_3446_MOESM1_ESM.tif]

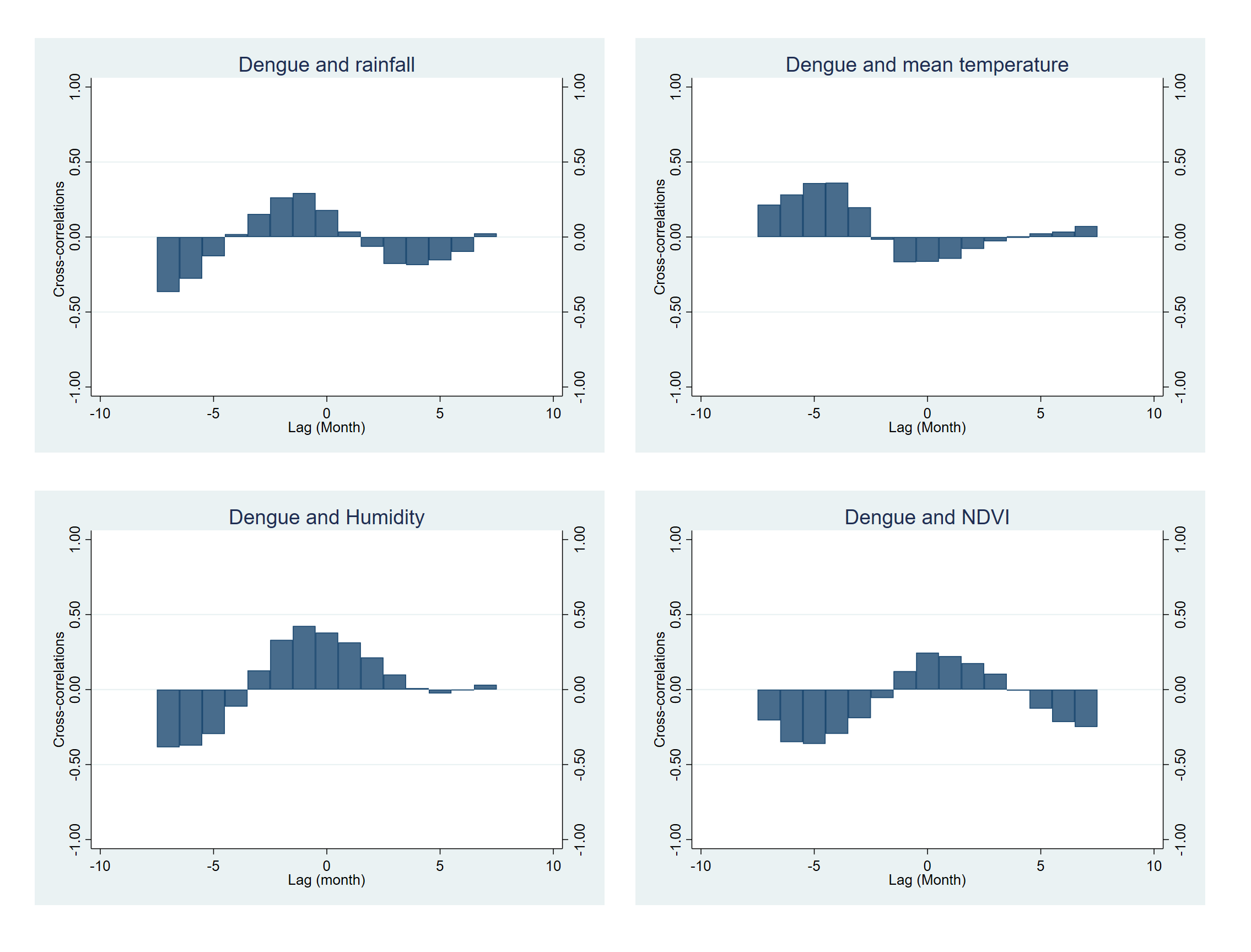

Supplement: Supplementary file 3 — Additional file 3: Figure S2. Cross-correlation analysis between dengue and rainfall, temperature humidity and NDVI, Cirebon, West Java, Indonesia. Cross-correlation analysis indicated a positive significant correlation between dengue with rainfall (lag 1–2 months), temperature (lag 4–6 months), humidity (lag 1–2 months) and NDVI (lag 0). [file 13071_2019_3446_MOESM3_ESM.tif]

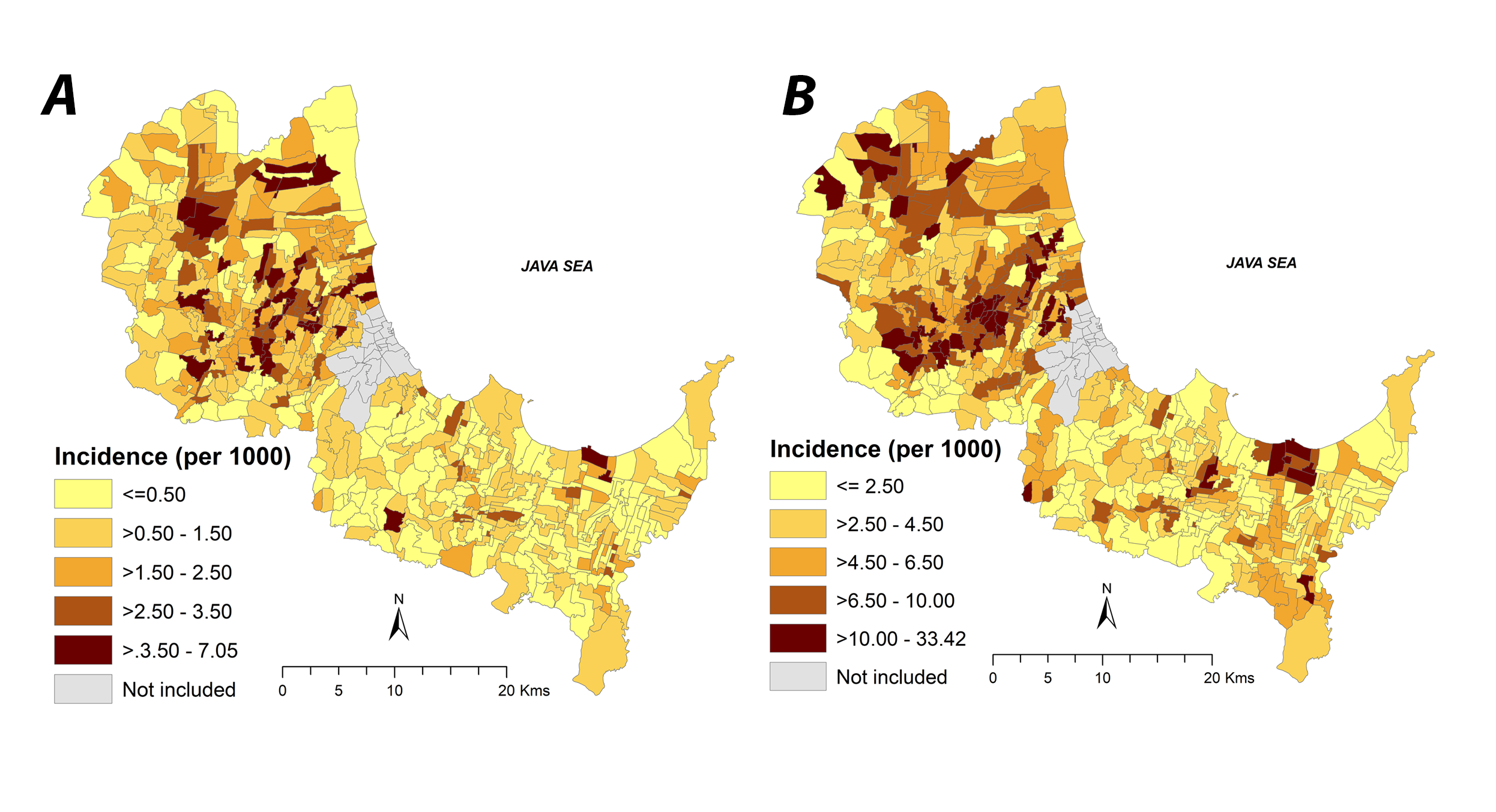

Supplement: Supplementary file 4 — Additional file 4: Figure S3. Crude incidence of dengue among U5s (A) and adolescents (5–19 years-old) (B) at village level, Cirebon, 2011–2017. [file 13071_2019_3446_MOESM4_ESM.tif]
