# Supplementary material for: Paediatric dengue infection in Cirebon, Indonesia: a temporal and spatial analysis of notified dengue incidence to inform surveillance
Source: Parasit Vectors. 2019 Apr 29;12:186. doi: 10.1186/s13071-019-3446-3 (PMC6489314; doi:10.1186/s13071-019-3446-3)
Supplement: Supplementary file 2 — Additional file 2: Table S1. Annual reported number of confirmed dengue cases by age-group, Cirebon district, 2011–2017. Table S2. Number of DHF, DSS, DD and suspected dengue cases in children, Cirebon district, 2011–2017. Table S3. Spearmanʼs correlation between covariates. Table S4. Models of associations between climatic factors and NDVI and dengue incidence as identified by a generalized linear model. Table S5. High-risk villages (n = 38) for dengue in children identified by LISA analysis, Cirebon district, 2011–2017. [file 13071_2019_3446_MOESM2_ESM.docx]

**Additional file 2**

**Table S1.** Annual reported number of confirmed dengue cases by age-group, Cirebon district, 2011-2017.

| **Year** | **Number of cases (%)** | | | **Total** |
| --- | --- | --- | --- | --- |
|  | **Under 5 yrs** | **5-19 yrs** | **>20 yrs** |  |
| 2011 | 26 | 90 | 74 | 190 |
| 2012 | 40 | 224 | 127 | 391 |
| 2013 | 94 | 531 | 214 | 839 |
| 2014 | 138 | 537 | 200 | 875 |
| 2015 | 222 | 868 | 170 | 1260 |
| 2016 | 352 | 1262 | 275 | 1889 |
| 2017 | 47 | 166 | 61 | 274 |
| Total | 919 | 3678 | 1121 | 5718 |

Note: this study was restricted to analyse dengue in children (≤19 yrs) (N=4597).

**Table S2.** Number of DHF, DSS, DD and suspected dengue cases in children, Cirebon district, 2011-2017.

| **Year** | **Number of cases by dengue classification (%)** | | | | **Total** |
| --- | --- | --- | --- | --- | --- |
|  | **DD** | **DHF** | **DSS** | **Suspect** |  |
| 2011 | 4 (2.84) | 104 (73.76) | 8 (5.67) | 25 (17.73) | 141 |
| 2012 | 5 (1.74) | 223 (77.70) | 36 (12.54) | 23 (8.01) | 287 |
| 2013 | 5 (0.73) | 568 (82.92) | 52 (7.59) | 60 (8.76) | 685 |
| 2014 | 8 (1.09) | 609 (83.31) | 58 (7.93) | 56 (7.66) | 731 |
| 2015 | 11 (0.96) | 993 (86.72) | 86 (7.51) | 55 (4.80) | 1145 |
| 2016 | 7 (0.42) | 1565 (92.82) | 42 (2.49) | 72 (4.27) | 1686 |
| 2017 | 0 (0.00) | 189 (62.17) | 24 (7.89) | 91 (29.93) | 304 |
| Total | 40 (0.80) | 4251 (85.38) | 306 (6.15) | 382 (7.67) | 4979 |

Note: In the analysis, we included only confirmed dengue cases (DD, DHF and DSS) (n=4597).

**Table S3**. Spearmanʼs correlation between covariates

| **Variables** | **Rainfall** | **RH** | **Temp** | **NDVI** |
| --- | --- | --- | --- | --- |
| Rainfall | 1.000 |  |  |  |
| RH | 0.863** | 1.000 |  |  |
| Temperature | -0.450** | -0.662** | 1.000 |  |
| NDVI | 0.126** | 0.356** | -0.423** | 1.000 |
|  |  |  |  |  |

Note: NDVI, indicate normalized difference vegetation index, * denotes P < 0.05; ** P < 0.01.

**Table S4.** Models of associations between climatic factors and NDVI and dengue incidence as identified by generalized linear model.

| **Model** | **Variables** | **AIC** | **BIC** |
| --- | --- | --- | --- |
| 1 | SAF+NDVI+Temp | 3426.95 | 3436.57 |
| 2 | SAF+NDVI_1_ | 3383.67 | 3390.93 |
| 3 | SAF+NDVI+Temp_1_ | 3351.15 | 3360.73 |
| 4 | SAF+Rain_1_ | 3339.12 | 3346.30 |
| 5 | SAF+NDVI_1_+Rain_1_ | 3324.77 | 3334.35 |
| 6 | SAF+NDVI_1_+Rain_1_+Temp_1_ | 3301.44 | 3313.29 |
| 7 | SAF+NDVI+Rain_1_+Temp_1_ | 3300.31 | 3312.15 |
| 8 | SAF+RH_0_ | 3207.50 | 3214.72 |
| 8 | SAF+Temp_2_ | 3173.30 | 3180.45 |
| 9 | SAF+NDVI_1_+Temp_2_ | 3167.61 | 3177.14 |
| 10 | SAF+NDVI_1_+Rain_3_+Temp+Rain_3_*Temp | 3115.90 | 3129.97 |
| 11 | SAF+NDVI_1_+Rain_1_+Temp_2_ | 3065.27 | 3077.06 |
| 12 | SAF+RH_0_+Temp2 | 3055.58 | 3065.21 |
| 13 | SAF+Temp_4_ | 2890.58 | 2897.66 |
| 14 | SAF+NDVI_1_+Temp_4_ | 2869.40 | 2878.83 |
| 15 | SAF+NDVI_1_+Rain_1_+Temp_4_ | 2851.78 | 2863.43 |
| 16 | SAF+RH0+Temp_4_ | 2713.66 | 2722.98 |
| 17 | SAF+NDVI1+RH0+Temp4 | 2675.97 | 2687.62 |
| 18* | SAF+Rain_2_+Temp_4_+RH_0_+NDVI_1_ | 2483.33 | 2497.08 |

Abbreviations: SAF, seasonality factor; NDVI, normalized difference vegetation index; Temp, monthly temperature; RH, relative humidity; AIC, Akaike information criteria; BIC, Bayesian information criteria. Subscripted number indicates lag (in month).

*selected as best-fit model

**Table S5.** High-risk villages (n=38) for dengue in children identified by LISA analysis, Cirebon district, 2011-2017

| **Kecamatan** | **Villages** |
| --- | --- |
| Sumber | Sendang |
| Palimanan | Cilukrak |
|  | Balerante |
|  | Beberan |
|  | Palimanan Timur |
|  | Pegagan |
| Plumbon | Lurah |
|  | Marikangen |
|  | Bodesari |
|  | Gombang |
|  | Karangmulya |
|  | Karangasem |
|  | Plumbon |
|  | Kebarepan |
|  | Pasanggrahan |
|  | Kedungsana |
| Depok | Kejuden |
|  | Waruroyom |
|  | Waruraya |
|  | Kasugenan Lor |
| Weru | Kertasari |
|  | Weru Kidul |
|  | Tegalwangi |
|  | Weru Lor |
| Plered | Panembahan |
|  | Gamel |
|  | Wotgali |
|  | Kaliwulu |
|  | Tegalsari |
| Klangenan | Serang |
| Jamblang | Jamblang |
|  | Wangunharja |
| Pangurangan | Pangurangan |
| Gempol | Palimanan Barat |
| Gegesik | Bayalangu Kidul |
|  | Bayalangu Lor |
|  | Gegesik Wetan |
|  | Gegesik Kidul |
